# Associations of Physical Fitness During Childhood with Arterial Structure and Stiffness in Adolescence: An 8-Year Follow-up Study

**DOI:** 10.1186/s40798-025-00841-w

**Published:** 2025-05-03

**Authors:** Emilia Laitinen, Sonja Soininen, Marja H. Leppänen, Katja Waller, Bert Bond, Niina Lintu, Avery D. Faigenbaum, Tomi Laitinen, Eero A. Haapala, Timo A. Lakka

**Affiliations:** 1 Faculty of Sport and Health Sciences, University of Jyväskylä, Jyväskylä, Finland; 2 Institute of Biomedicine, School of Medicine, University of Eastern Finland, Kuopio Campus, Kuopio, Finland; 3 Physician and Nursing Services, Health and Social Services Centre, Wellbeing Services County of North Savo, Varkaus, Finland; 4 Faculty of Medicine, University of Helsinki, Helsinki, Finland; 5 Faculty of Sport and Health Sciences, University of Exeter, Exeter, UK; 6 Department of Kinesiology and Health Sciences, The College of New Jersey, Ewing Township, NJ USA; 7 Department of Clinical Physiology and Nuclear Medicine, Kuopio University Hospital, Kuopio, Finland; 8 Kuopio Research Institute of Exercise Medicine, Kuopio, Finland

**Keywords:** Cardiorespiratory fitness, Muscular fitness, Motor fitness, Arterial stiffness, Atherosclerosis, Cardiovascular disease, Children, Adolescents

## Abstract

**Background:**

Cardiovascular diseases (CVDs) are the leading cause of morbidity and premature mortality globally. While the relationship between indicators of physical fitness and arterial structure and stiffness are reasonably well-studied in adults, these associations in children and adolescents remain less understood. The aim of this study was to investigate longitudinal associations of cardiorespiratory fitness, muscular fitness and motor fitness with arterial structure and stiffness from childhood to adolescence.

**Results:**

Higher mean value of VO_2peak_/LM from childhood to adolescence was associated with higher carotid intima-media thickness (cIMT) at 8-year follow-up (β = 0.184, 95% confidence interval [CI] = 0.019 to 0.350). Better performance in sit-up test at baseline was associated with lower cardio-ankle vascular index (CAVI) (β = − 0.219, 95% CI = − 0.387 to − 0.051) at 8-year follow-up, and higher mean sit-up performance from baseline to 8-year follow-up was associated with lower carotid-femoral pulse-wave velocity (cfPWV) (β = − 0.178, 95% CI = − 0.353 to − 0.003) and CAVI (β = − 0.190, 95% CI = − 0.365 to − 0.016) at 8-year follow-up. Also cross-sectionally, better sit-up performance at 8-year follow-up was associated with lower cfPWV (β = − 0.232, 95% CI = − 0.411 to − 0.054) and CAVI (β = − 0.185, 95% CI = − 0.365 to − 0.005) and higher carotid artery distensibility (β = 0.165, 95% CI = 0.004 to 0.327) at 8-year follow-up. Most of the associations were explained by body fat percentage (BF%).

**Conclusions:**

Physical fitness had a weak if any association with indicators of arterial structure and arterial stiffness in adolescence. BF% largely explained the associations of higher VO_2peak_/LM with higher cIMT and better sit-up performance with lower arterial stiffness in adolescents. Therefore, preventing adiposity rather than improving CRF should be addressed in public health strategies to prevent CVDs in general paediatric populations.

**Key Points:**

- Better sit-up performance was associated with lower arterial stiffness, but the association was largely explained by body fat percentage.
- Lower body muscular strength, handgrip strength, or motor fitness was not associated with arterial stiffness or carotid artery intima-media thickness.
- Measures other than cardiorespiratory fitness, muscular fitness, or motor fitness, such as adiposity, should be used to screen children and adolescents at increased risk of cardiovascular diseases.

## Background

Cardiovascular diseases (CVDs) are the leading cause of morbidity and pre-mature mortality worldwide [1, 2]. Even though the clinical complications of CVDs are not usually detectable until adulthood, the pathological processes leading to CVDs begin early in life [3]. The early signs of atherosclerosis, such as increased arterial stiffness assessed by pulse wave velocity (PWV) and carotid artery intima-media thickness (cIMT), also predict future clinical CVD events in adults [4, 5] such as left ventricular hypertrophy [6], heart failure, myocardial ischemia and even myocardial infarction [7]. Therefore, to reduce the risk of developing CVDs throughout the life course, the early identification of individuals who may develop CVDs later in life is essential. Early arterial ageing can be detected by assessing arterial stiffness in youth, and by monitoring cIMT, adaptive remodelling of the arteries can be discovered [7]. However, procedures for assessing arterial stiffness or cIMT are not commonly available at paediatric clinics or school health care centres due to the high cost of the equipment and the lack of appropriately trained staff. Therefore, easily assessed indicators of early atherosclerosis for identifying youth at increased risk for CVD later in life are needed.

Better muscular fitness and motor fitness have become recognized for their possible health benefits such as lower levels of obesity and cardiometabolic risk and higher bone mineral density [8]. Compared to CRF testing, muscular fitness and motor fitness testing such as handgrip strength tests and standing long-jump tests are practical to administer in most settings without expensive devices [9]. Therefore, physical fitness testing has been considered a valuable tool for evaluating health status and CVD risk in children and adolescents [10, 11]. Physical fitness is a set of health- and skill-related attributes, including cardiorespiratory fitness (CRF), muscular fitness and motor fitness [12, 13]. CRF is defined as the capacity of cardiovascular system to deliver oxygen to the working skeletal muscles and the ability of working muscles to use oxygen in energy production to support locomotion [13], muscular fitness describes the status of muscular strength and endurance [14], and motor fitness consists of agility, balance and speed [15].

CRF is considered one of the most accurate indicators of physical health status [10]. Therefore, measuring CRF is a valuable assessment of CVD risk in youth [16]. Lower CRF has been independently associated with several cardiovascular risk factors including increased arterial stiffness [17], adiposity, insulin resistance and dyslipidaemia in youth [7, 18]. Moreover, low CRF has been independently associated with increased arterial stiffness and impaired arterial dilatation capacity among children in cross-sectional studies [19–21]. Lower CRF has also been found to predict a lower brachial artery distensibility and higher carotid-radial PWV one year later in children [22]. In contrast, we have previously reported no associations of CRF with arterial stiffness and dilatation capacity over a 2-year follow-up period in children [23]. Moreover, the overall evidence on the associations of CRF with the indicators of arterial structure and arterial stiffness in children is inconsistent and mostly based on cross-sectional studies [24–26]. In this study, we use cIMT as a measure of arterial structure and cfPWV, cardio-ankle vascular index (CAVI) and carotid artery distensibility (CAD) as measures of arterial stiffness.

Muscular and motor fitness testing such as sit-up and handgrip tests are relatively time and space-efficient making them suitable for clinical use in healthcare settings with limited time and space resources. While poorer muscular fitness has been associated with insulin resistance, dyslipidaemia and systemic low-grade inflammation in children and adolescents [27, 28], few studies have investigated the associations of muscular fitness measured by a handgrip test with indicators of arterial structure and stiffness, such as cIMT and PWV, in youth [24]. In a small cross-sectional study in children aged 6–11 years, handgrip strength was not associated with PWV [29]. In contrast, higher handgrip strength has been associated with lower cIMT in children aged 11–12 years [30]. However, there are no previous studies investigating whether muscular and motor fitness in childhood predicts arterial structure or stiffness in adolescence. Furthermore, a pertinent question of whether selected and widely used indicators of muscular fitness and motor fitness can be used to screen children at increased risk of CVDs remains unanswered because most of the previous studies have assessed muscular fitness and motor fitness using only handgrip strength testing.

Additional research is required to investigate if cardiorespiratory fitness, muscular fitness and motor fitness assessed during childhood associate with indicators of arterial structure or stiffness later in life [31]. Therefore, this study examined the associations of physical fitness indicators (peak oxygen uptake per lean mass, maximal workload per lean mass, handgrip strength, standing long jump performance, sit-up performance, and shuttle run performance) in childhood with the measures of arterial structure and stiffness including cIMT, cfPWV, CAVI and CAD in adolescence. Cross-sectional associations in adolescence were also examined using the same indicators as in longitudinal analysis. Secondly, we studied the associations of changes in the indicators of CRF, muscular fitness and motor fitness from childhood to adolescence with arterial structure and stiffness indicators in adolescence. Finally, we examined whether the means of the indicators of physical fitness from childhood to adolescence were associated with the indicators of arterial structure and stiffness in adolescence.

## Methods

### Study Design and Participants

The present data are from the Physical Activity and Nutrition in Children (PANIC) Study (ClinicalTrials.gov NCT01803776), which is an 8-year physical activity and dietary intervention study and a long-term follow-up study in a population sample of children from the city of Kuopio, Finland. The Research Ethics Committee of the Hospital District of Northern Savo approved the study protocol in 2006 (Statement 69/2006) and in 2015 (Statement 422/2015). The caregivers of the children gave their written parental permission, and the children provided their assent to participate at baseline and 2-year follow-up, and the caregivers and the adolescents provided their written consent at the 8-year follow-up. The PANIC study has been carried out in accordance with the principles of the Declaration of Helsinki as revised in 2008.

A total of 736 children aged 6–9 years were invited to participate in the baseline examination in 2007–2009. Altogether, 512 children attended the baseline examinations, and 504 children had no exclusion criteria for participating in the intervention study. The 2-year follow-up examinations were conducted for 438 participants at the age of 9–11 years in 2009–2011 and the 8-year follow-up examinations for 277 participants at the age of 15–17 years in 2016–2017. The participants included in the present analyses did not differ in age, sex distribution, pubertal status, body mass index standard deviation score, or body fat percentage (BF%) at the 8‐year follow‐up from those excluded from the analyses (*p* > 0.10). Those who did not participate in the 8-year follow-up examinations had higher BF% at baseline (mean difference 2.4, 95% confidence interval [CI] = 0.75 to 4.01) and were more likely to have parents without university-level education at baseline (27% vs. 43%; *p* < 0.001) than those who attended the 8-year follow-up.

### Assessments of Physical Fitness

CRF was assessed using an incremental cycle ergometer exercise test until exhaustion, muscular fitness using the sit-up, standing long jump, and handgrip strength tests, and motor fitness using the 10 × 5 m shuttle run test at baseline, 2-year follow-up, and 8-year follow-up. CRF was quantified as power output per lean body mass (W_max_/LM). At the 2-year and 8-year follow-ups, the incremental cycle ergometer test was coupled with respiratory gas analysis and thus allowed the use of peak oxygen uptake per lean body mass (VO_2peak_/LM) as an indicator of CRF. The indicators of arterial structure and arterial stiffness were assessed only at the 8-year follow-up.

#### Assessment of Body Size and Composition

Body mass was measured twice to an accuracy of 0.1 kg, with the children having fasted for 12 h, emptied the bladder, and standing in light underwear using a weight scale integrated into a calibrated InBody® 720 bioelectrical impedance device (Biospace, Seoul, South Korea). The mean of these two values was used in the statistical analyses. Height was measured three times with the children standing in the Frankfurt plane without shoes using a wall-mounted stadiometer to an accuracy of 0.1 cm. The mean of the nearest two values was used in the statistical analyses. Lean body mass (LM) and BF% were measured by the Lunar® dual-energy X-ray absorptiometry device (GE Medical Systems, Madison, WI, USA) using a standardised protocol [32]. Body mass index (BMI) was calculated by dividing weight (kg) by height (m) squared. Body mass index-standard deviation score (BMI-SDS) was calculated based on the Finnish reference data [33].

#### Assessment of CRF

CRF was assessed by a maximal ramp exercise test with an electromagnetically braked Ergoselect 200 K bicycle ergometer (Ergoline, Bitz, Germany) at the baseline, 2-year follow-up, and 8-year follow-up. The exercise test protocol included a 2.5 min period sitting on the ergometer, a 3 min warm-up period at 5 Watts (W), a 1 min steady-state period at 20 W, an exercise period with workload increases of 1 W every 6 s until voluntary exhaustion and a 4 min cooling-down period at 5 W. The participants were asked to keep the cadence of 70–80 rounds per minute during the test. They were verbally encouraged to exercise until voluntary exhaustion and the test was terminated when the participant was unable to keep the cadence of 50 or required to stop the test. The CRF test protocol was identical at the baseline, 2-year follow-up, and 8-year follow-up. The protocol was considered valid at all time points since Midgley and colleagues [34] suggested that to elicit valid VO_2peak_ values, the duration of the cycle ergometer tests can range from 7 to 26 min.

CRF was assessed by W_max_/LM at the baseline, 2-year follow-up, and 8-year follow-up and additionally by VO_2peak_/LM at the 2-year and 8-year follow-up. Respiratory gases were collected by the breath-by-breath method from the anticipatory period of sitting on the bicycle ergometer to the post-exercise rest using paediatric masks (Hans-Rudolph, Shawnee, USA), and were analysed using the Oxycon Pro® respiratory gas analyser (Jaeger, Hoechberg, Germany). The analyser was calibrated according to the manufacturer’s instructions before each test. VO_2peak_ was defined as the highest 15 s average value recorded during the last minute of the test.

#### Assessment of Muscular Fitness

Abdominal muscular strength and endurance were assessed by the sit-up test. The children were asked to lie on their back with knees flexed at 90°, feet positioned on the ground and arms kept behind the neck. The children were told to do as many sit-ups as possible in 30 s with the elbows coming up to touch the knees with the assistant holding their feet on the floor. The test score was the number of technically correct sit-ups completed in 30 s [35]. The sit-up tests have been found to have acceptable reproducibility [36] and reliability, with higher values for reliability reported for adolescents compared to children [37].

Lower body muscular strength was assessed by the standing long jump test. The children were asked to stand with their feet together, jump as far as possible and land on both feet. The test score was the longest jump of three attempts in centimetres. The standing long jump test has been found to have acceptable reproducibility in youth [35].

Handgrip strength was assessed by the Martin vigorimeter (Martin, Tuttlingen, Germany). The children were asked to keep the elbow close to the body with the arm flexed at 90° and then squeeze a rubber bulb as hard as possible. The test was performed three times with each hand and the mean of the best result of both hands was used in the statistical analyses. Handgrip strength was expressed in kilopascals (kPa) and scaled by LM. The handgrip strength test has been found to have acceptable reproducibility in youth [36].

#### Assessment of Motor Fitness

Speed and agility were assessed by the 10 × 5 m shuttle run test. The children were asked to run five metres from a starting line to another line as quickly as possible, turn quickly on the line, run back to the starting line, and continue until five shuttles were completed as quickly as possible. The test score was the running time in seconds, with a longer time indicating a poorer performance. The test was performed once. The 10 × 4 m shuttle run test has been found to be reliable with an intraclass correlation of 0.69 between the measurements taken one week apart, and the 10 × 4 m speed and agility shuttle run test has been reported to have moderate to good reproducibility with a 0.1 s inter-trial difference [38].

### Assessment of Arterial Structure and Stiffness

#### cfPWV and CAVI

cfPWV and CAVI were measured using the CircMon® B202 impedance cardiography device (JR Medical Ltd., Saku Vald., Estonia) after a 15 min rest in the supine position. The distal impedance plethysmography was recorded from a popliteal artery at the knee joint level. The CircMon software estimates the foot of the impedance cardiography signal that coincides with pulse transmission in the aortic arch and the foot of the impedance plethysmography signal that coincides with pulse transmission in the popliteal artery. The CircMon device has been found to be valid and reproducible in the assessment of cfPWV [39, 40].

The CircMon software calculated cfPWV (m/s) by dividing the assessed distance (L) by utilising the measured pulse transit time (Δt). The CAVI parameters were calculated as β = 2 ρ x ln/(Ps–Pd) x ln (Ps/Pd) x PWV^2^, where ρ is estimated blood density [41], Ps is systolic blood pressure (SBP) in mmHg, Pd is diastolic blood pressure (DBP) in mmHg, and cfPWV is assessed from the origin of the aorta to the tibial artery at the ankle through the femoral artery [42].

#### Carotid IMT and CAD

Trained sonographers assessed IMT and the elasticity of the left common carotid artery utilising the Acuson Sequoia 512 Ultrasound Mainframe® (Acuson, Mountain View, CA, USA) with a 14.0 MHz linear array transducer using a standardised protocol [43]. The sonographers analysed the ultrasound scans offline from the digitally stored images. Three measurements of the far wall at end-diastole were taken to derive maximal cIMT. For the assessments of carotid artery elasticity, the diameter of the common carotid artery at end-diastole and end-systole was measured at least twice. In addition, the sonographer measured SBP and DBP from the brachial artery just before and directly after the ultrasound scans. The means of the end-diastolic and end-systolic diameters as well as SBP and DBP values were used to calculate CAD as ([systolic diameter-diastolic diameter]/diastolic diameter)/(SBP-DBP). These measurements and analyses were performed at the Department of Clinical Physiology and Nuclear Medicine, Kuopio University Hospital.

### Assessment of Pubertal Status

A research physician assessed pubertal status according to breast development in girls (stages M1-5) and testicular volume by an orchidometer in boys (stages G1-5) as described by Marshall and Tanner [44, 45].

### Statistical Methods

The data were analysed using the SPSS for Windows software version 28.0 (IBM corp., Armonk, NY, USA). Before the analysis, the missing data on pubertal status and VO_2peak_/LM were replaced using multiple imputations, using 20 imputed data sets. The differences in boys and girls at the baseline and the indicators of arterial structure and stiffness at the 8-year follow-up were investigated with the Student’s t test, the Mann–Whitney U test, or the Chi-square test.

The associations of the indicators of physical fitness at the baseline, 2-year follow-up and 8-year follow-up with the indicators of arterial structure and stiffness at the 8-year follow-up were investigated by linear regression analyses adjusted for age and sex at the 8-year follow-up. If the associations were statistically significant after adjustment for age and sex, the data were further adjusted for other potential confounders, including pubertal status, BF% or SBP, which were entered into the models one by one with age and sex. To investigate whether the associations of physical fitness at the baseline and 2-year follow-up with arterial structure and stiffness at the 8-year follow-up were independent of the corresponding indicators of physical fitness at the 8-year follow-up, the data were further adjusted for these indicators of physical fitness at the 8-year follow-up.

Thereafter, we investigated the associations of changes in the indicators of physical fitness from baseline to the 8-year follow-up (W_max_/LM_8-year-follow-up_—W_max_/LM_baseline_, muscular fitness_8-year follow-up_—muscular fitness_baseline_, motor fitness_8-year follow-up_—motor fitness_baseline_) or from the 2-year follow-up to the 8-year follow-up (VO_2peak_/LM_8-year-follow-up_—VO_2peak_/LM_2-year follow-up_) with the indicators of arterial structure and stiffness at the 8-year follow-up adjusted for age and sex at the 8-year follow-up. If the associations were statistically significant after adjustment for age and sex, the data were further adjusted for other potential confounders, including pubertal status, BF% or SBP, which were entered into the models one by one with age and sex. The change in VO_2peak_/LM was computed only from the 2-year follow-up to the 8-year follow-up because VO_2peak_/LM was not assessed at baseline. Finally, we investigated the associations of the means of the indicators of physical fitness from the baseline, 2-year follow-up, and 8-year follow-up with the indicators of arterial structure and stiffness at the 8-year follow-up adjusted for age and sex at the 8-year follow-up. If the associations were statistically significant after adjustment for age and sex, the data were further adjusted for other potential confounders, including pubertal status, BF% or SBP, which were entered into the models one by one with age and sex. The mean values from childhood to adolescence were computed as (muscular fitness_baseline_ + muscular fitness_2-year follow-up_ + muscular fitness_8-year follow-up_)/3, (motor fitness_baseline_ + motor fitness_2-year follow-up_ + motor fitness_8-year follow-up_)/3, (W_max_/LM_baseline_ + W_max_/LM_2-year follow-up_ + W_max_/LM_8-year follow-up_)/3 and (VO_2peak_/LM_2-year follow-up_ + VO_2peak_/LM_8-year follow-up_)/2 adjusted for age and sex at the 8-year follow-up. The data were corrected for multiple comparisons using the Benjamini–Hochberg false discovery rate (FDR) procedure using the FDR value of 0.2 (FDR0.2). If the associations were statistically significant after adjustment for age and sex, the data were further adjusted for other potential confounders, including pubertal status, BF% or SBP, which were entered into the models one by one with age and sex.

## Results

### Participants

The participants did not differ in sex distribution, age, or BMI-SDS from all children who started the first grade in 2007–2009 based on data from the standard school health examinations performed for all Finnish children before the first grade (data not shown). The data on the variables used in the statistical analyses for the associations of CRF, muscular fitness, and motor fitness with cIMT and CAD were available for 160 children and the data on the variables used in the statistical analyses for the associations of these indicators of physical fitness with cfPWV and CAVI were available for 131 children.

Table 1 describes the basic characteristics and Table 2 the indicators of physical fitness and arterial structure and stiffness of the participants at the 8-year follow-up.

**Table 1:** Basic characteristics at the 8-year follow-up

	All (= 160)	Girls (= 76)	Boys (= 84)
Age (years)	15.8 (0.4)	15.7 (0.4)	15.9 (0.5)
Pubertal status (*n*)			
Stage 3^ab^	9	3	6
Stage 4^ab^	95	43	52
Stage 5^ab^	49	27	22
Height (cm)	171.6 (8.4)	166.1 (5.7)	176.6 (7.2)
Weight (kg)	62.7 (12.1)	59.0 (8.8)	66.0 (13.7)
Body fat mass (kg)	14.7 (9.0)	17.6 (7.1)	12.1 (9.7)
Body fat percentage (%)	22.7 (10.3)	29.2 (7.2)	16.8 (9.0)
Lean body mass (kg)	45.7 (8.4)	38.8 (3.7)	51.8 (6.5)
Body mass index—standard deviation score	0.04 (0.96)	0.17 (0.82)	− 0.08 (1.06)

Displayed as means (SD). ^a^N = 153. ^b^ = pubertal status described by Tanner

**Table 2:** Indicators of physical fitness and arterial structure and stiffness at the 8-year follow-up

	All (= 160)	Girls (= 76)	Boys (= 84)
VO_2peak_ (ml/min)^a^	2857 (600)	2384 (329)	3257 (473)
VO_2peak_/LM (ml/min/LM)^a^	62.10 (5.94)	61.41 (4.94)	62.68 (6.64)
Maximal power output/kg of body mass (W_max_/kg)	3.19 (0.63)	2.94 (0.45)	3.42 (0.68)
Maximal power output/LM (W_max_/LM)	4.41 (0.53)	4.47 (0.45)	4.36 (0.60)
Handgrip (kPa)	105.6 (23.0)	97.1 (16.6)	113.3 (25.3)
Standing long jump (cm)	192.4 (30.0)	173.0 (21.8)	210.0 (25.1)
Sit-up (*n*/30 s)	22.1 (5.6)	20.1 (4.6)	23.8 (5.8)
Shuttle-run (s)	20.6 (1.9)	21.4 (1.5)	19.8 (2.0)
Diastolic blood pressure (mmHg)	68 (10)	67 (10)	68 (10)
Systolic blood pressure (mmHg)	114 (10)	112 (9)	115 (11)
Pulse wave velocity (m/s)	5.86 (0.67)	5.90 (0.68)	5.82 (0.65)
Carotid-ankle vascular index	6.6 (1.8)	6.8 (2.0)	6.3 (1.5)
Carotid intima media thickness (mm)	0.45 (0.06)	0.43(0.05)	0.46 (0.07)
Carotid artery distensibility (%/10 mmHg)	2.8 (0.7)	2.9 (0.8)	2.7 (0.7)
Time to exercise cessation (min:sec)^b^	19:03 (4:17)	19:35 (4:02)	18:25 (4:33)

Displayed as means (SD). ^a^N = 142. VO_2peak_ = peak oxygen uptake. LM = kgs lean mass. W = watts. kPa = kilopascal. ^b^ = Minutes and seconds from start of the cycle ergometer test to the exercise cessation

### Longitudinal Associations of Physical Fitness at the Baseline and 2-Year Follow-up with Arterial Structure and Stiffness at 8-Year Follow-up

A higher number of sit-ups at baseline was associated with a lower CAVI at the 8-year follow-up after adjustment for age and sex at the 8-year follow-up (Table 3). This association remained statistically significant after further adjustment for SBP (standardized regression coefficient β = − 0.220, 95% CI = − 0.390 to − 0.050), BF% (β = − 0.207, 95% CI = − 0.379 to − 0.035) or pubertal status (β = − 0.203, 95% CI = − 0.380 to − 0.030) at 8-year follow-up. However, the association did not remain significant after the FDR0.2 correction (*p* = 0.290). Other indicators of CRF, muscular fitness and motor fitness at baseline were not associated with those of arterial structure or stiffness at the 8-year follow-up. Indicators of physical fitness at the 2-year follow-up were not associated with the indicators of arterial structure or stiffness at the 8-year follow-up (Table 3).

**Table 3:** Associations of indicators of physical fitness at the baseline, 2-year follow-up and 8-year follow-up with the indicators of arterial structure and stiffness at 8-year follow-up

	Pulse wave velocity β (95% CI)	Cardio-ankle vascular index β (95% CI)	Carotid intima media thickness β (95% CI)	Carotid artery distensibility β (95% CI)
*Baseline*				
Maximal workload/LM (W_max_/LM)	− 0.015 (− 0.193 to 0.164)	− 0.123 (− 0.300 to 0.054)	0.050 (− 0.106 to 0.207)	− 0.010 (− 0.168 to 0.147)
Handgrip strength/LM (kPa/LM)	0.064 (− 0.110 to 0.239)	− 0.037 (− 0.212 to 0.138)	0.011 (− 0.147 to 0.168)	0.038 (− 0.120 to 0.196)
Standing long jump (cm)	− 0.124 (− 0.308 to 0.059)	− 0.128 (− 0.311 to 0.055)	− 0.078 (− 0.240 to 0.083)	0.037 (− 0.125 to 0.200)
Sit-up (number of repetitions/30 s)	− 0.141 (− 0.312 to 0.030)	− **0.219 (**− **0.387 to **− 0.051)*	0.066 (− 0.088 to 0.219)	− 0.021 (− 0.175 to 0.134)
10 × 5 m shuttle run (time, s)	0.099 (− 0.076 to 0.274)	0.139 (− 0.034 to 0.313)	0.010 (− 0.147 to 0.167)	− 0.126 (− 0.283 to 0.030)
*2-year follow-up*				
VO_2peak_/LM (ml/min/LM)^a^	0.033 (− 0.148 to 0.213)	− 0.018 (− 0.201 to 0.165)	0.150 (− 0.026 to 0.326)	0.027 (− 0.137 to 0.191)
Maximal workload/LM (W_max_/LM)	− 0.018 (− 0.198 to 0.162)	− 0.052 (− 0.232 to 0.127)	0.128 (− 0.028 to 0.284)	0.016 (− 0.141 to 0.174)
Handgrip strength/LM (kPa/LM)	0.084 (− 0.087 to 0.255)	− 0.070 (− 0.241 to 0.102)	− 0.009(− 0.162 to 0.144)	− 0.089 (− 0.243 to 0.064)
Standing long jump (cm)	− 0.142 (− 0.322 to 0.038)	− 0.068 (− 0.248 to 0.113)	− 0.081 (− 0.244 to 0.081)	− 0.036 (− 0.200 to 0.128)
Sit-up (number of repetitions/30 s)	− 0.016 (− 0.189 to 0.158)	− 0.016 (− 0.190 to 0.157)	− 0.021 (− 0.176 to 0.134)	− 0.013 (− 0.169 to 0.143)
10 × 5 m shuttle run (time, s)	− 0.023 (− 0.198 to 0.152)	− 0.016 (− 0.191 to 0.159)	0.021 (− 0.136 to 0.178)	0.007 (− 0.151 to 0.165)
*8-year follow-up*				
VO_2peak_/LM (ml/min/LM)^a^	− 0.021 (− 0.216 to 0.174)	0.055 (− 0.131 to 0.240)	0.135 (− 0.028 to 0.298)	0.075 (− 0.095 to 0.246)
Maximal workload/LM (W_max_/LM)	− 0.156 (− 0.326 to 0.015)	− 0.078 (− 0.250 to 0.093)	0.062 (− 0.092 to 0.216)	0.093 (− 0.061 to 0.247)
Hand grip strength/LM (kPa/LM)	− 0.072 (− 0.253 to 0.109)	− 0.171 (− 0.350 to 0.007)	0.105 (− 0.057 to 0.266)	0.089 (− 0.074 to 0.251)
Standing long jump (cm)	− 0.130 (− 0.345 to 0.086)	− 0.048 (− 0.264 to 0.168)	0.000 (− 0.196 to 0.195)	− 0.013 (− 0.209 to 0.182)
Sit-up (number of repetitions/30 s)	− **0.232 (**− **0.411 to **− 0.054)^*^	− **0.185 (**− **0.365 to **− 0.005)^*^	− 0.017 (− 0.180 to 0.146)	0.165 (0.004 to 0.327)^*^
10 × 5 m shuttle run (time, s)	0.125 (− 0.061 to 0.311)	0.145 (− 0.041 to 0.330)	− 0.012 (− 0.182 to 0.157)	0.028 (− 0.142 to 0.198)

Data are standardised regression coefficients (95% confidence intervals) from linear regression models adjusted for age and sex at 8-year follow-up. *p < 0.05. Standardised regression coefficients (95% confidence intervals) and *p*-values for statistically significant associations are bolded. W = watts. LM = kgs of lean mass. kPa = kilopascal. VO_2peak_ = peak oxygen uptake. ^a^ = VO_2peak_/LM was not measured at baseline

### Cross-sectional Associations of Physical Fitness with Arterial Structure and Stiffness at the 8-Year Follow-up

A higher number of sit-ups was cross-sectionally associated with a lower cfPWV and CAVI, as well as higher CAD after adjustment for age and sex at the 8-year follow-up (Table 3). The associations of sit-up performance with cfPWV (β = − 0.228, 95% CI = − 0.407 to − 0.049) and CAVI (β = − 0.185, 95% CI = − 0.368 to − 0.002) but not with CAD (β = 0.155, 95% CI = − 0.001 to 0.311), remained statistically significant after further adjustment for SBP. The associations of sit-up performance with cfPWV (β = − 0.147, 95% CI = − 0.343 to 0.050), CAVI (β = − 0.167, 95% CI = − 0.369 to 0.034) and CAD (β = 0.139, 95% CI = − 0.043 to 0.321) did not remain statistically significant after further adjustment for BF% at the 8-year follow-up. The association of sit-up performance with cfPWV (β = − 0.224, 95% CI = − 0.410 to − 0.038), but not with CAVI (β = − 0.158, 95% CI = − 0.346 to 0.029) and CAD (β = 0.134, 95% CI = − 0.032 to − 0.300), remained statistically significant after additional adjustment for pubertal status at the 8-year follow-up. Other indicators of physical fitness were not cross-sectionally associated with the indicators of arterial structure or stiffness at the 8-year follow-up. The inverse association of sit-up performance with cfPWV remained significant after the FDR 0.2 correction (*p* = 0.154). However, the associations of sit-up performance with lower CAVI (*p* = 0.290) and higher CAD (*p* = 0.608) did not remain significant after the FDR 0.2 correction.

### Associations of Changes and Means of Physical Fitness from the Baseline to the 8-Year Follow-up with Arterial Structure and Stiffness at the 8-Year follow-up

Changes in the indicators of physical fitness from baseline to the 8-year follow-up were not associated with the indicators of arterial structure or stiffness at the 8-year follow-up after adjustment for age and sex at the 8-year follow-up (data not shown). However, a higher mean of VO_2peak_/LM from the 2-year and 8-year follow-up was associated with a higher cIMT at the 8-year follow-up adjusted for age and sex (Table 4). This association remained statistically significant after further adjustment for pubertal status (β = 0.181, 95% CI = 0.004 to 0.350) but not SBP (β = 0.159, 95% CI = − 0.011 to 0.321) or BF% (β = 0.154, 95% CI = − 0.013 to 0.314). The association did not remain significant after the FDR 0.2 correction (*p* = 0.632). Furthermore, a higher mean of the number of sit-ups from the baseline, 2-year follow-up, and 8-year follow-up was associated with lower cfPWV and CAVI adjusted for age and sex (Table 4). These associations of the mean sit-up performance from the baseline, 2-year follow-up, and 8-year follow-up with cfPWV and CAVI remained statistically significant after further adjustment for SBP (for cfPWV β = − 0.176, 95% CI = − 0.315 to − 0.001, for CAVI β = − 0.190, 95% CI = − 0.367 to − 0.013) but not BF% (for cfPWV β = − 0.095, 95% CI = − 0.282 to 0.093, for CAVI β = − 0.174, 95% CI = − 0.364 to 0.017) or pubertal status (for cfPWV β = − 0.175, 95% CI = − 0.359 to 0.007, for CAVI β = − 0.166, 95% CI = − 0.350 to 0.015). Other mean values of the indicators of physical fitness were not associated with the indicators of arterial structure or stiffness at the 8-year follow-up (Table 4).

**Table 4:** Associations of means of indicators of physical fitness from the baseline, 2-year follow-up, and 8-year follow-up with indicators of arterial structure and stiffness at 8-year follow-up

	Pulse wave velocity β (95% CI)	Cardio-ankle vascular index β (95% CI)	Carotid intima media thickness β (95% CI)	Carotid artery distensibility β (95% CI)
Baseline to 8-year follow-up				
VO_2peak_/LM (ml/min/LM)^a^	0.008 (− 0.177 to 0.193)	0.024 (− 0.159 to 0.206)	0.184 (0.019 to 0.350)^*^	0.066 (− 0.098 to 0.230)
Maximal workload (W/LM)	− 0.087 (− 0.261 to 0.088)	− 0.108 (− 0.282 to 0.066)	0.077 (− 0.244 to 0.089)	0.132 (− 0.034 to 0.298)
Hand grip strength (kPa/LM)	0.030 (− 0.145 to 0.205)	− 0.123 (− 0.296 to 0.051)	0.048 (− 0.110 to 0.205)	0.012 (− 0.146 to 0.170)
Standing long jump (cm)	− 0.159 (− 0.358 to 0.040)	− 0.093 (− 0.293 to 0.108)	− 0.062 (− 0.243 to 0.118)	− 0.008 (− 0.189 to 0.173)
Sit-up (number of repetitions/30 s)	− **0.178 (**− **0.353 to –0.003**)^*^	− **0.190 (**− 0.365 to –0.016)^*^	0.013 (− 0.145 to 0.171)	0.063 (− 0.095 to 0.222)
10 × 5 m shuttle run (time, s)	0.090 (− 0.092 to 0.271)	0.120 (− 0.061 to 0.301)	0.009 (− 0.156 to 0.173)	− 0.051 (− 0.215 to 0.114)

The data are standardised regression coefficients (95% confidence intervals) from linear regression models adjusted for age and sex at 8-year follow-up. Standardised regression coefficients (95% confidence intervals) and *p*-values for statistically significant associations are bolded. *p < 0.05. Baseline to 8-year follow-up describes time points used to calculate the means of indicators of physical fitness. W = watts. LM = kgs of lean mass. kPa = kilopascal. VO_2peak_ = peak oxygen uptake. ^a^ = VO_2peak_/LM was not measured at baseline

## Discussion

We found that higher mean VO_2peak_/LM, indicating better average cardiorespiratory fitness, in childhood and adolescence was associated with higher cIMT but not with the indicators of arterial stiffness in adolescence. Moreover, better sit-up performance, indicating higher abdominal muscular strength and endurance, in childhood was associated with lower CAVI in adolescence. Higher mean sit-up performance in childhood and adolescence was also associated with lower cfPWV and CAVI in adolescence. In addition, better sit-up performance in adolescence was associated with lower cfPWV and CAVI, as well as higher CAD in adolescence. These associations were partly explained by confounding factors such as BF% and pubertal status. The indicators of lower body muscular strength, handgrip strength, and motor fitness were not associated with arterial structure or stiffness.

In line with the results of some previous studies, we found that W_max_/LM was not associated with carotid IMT [46, 47]. However, some studies have reported inverse associations of CRF with PWV [21] and aortic IMT [46] in children and adolescents. Differences in the assessment and scaling of CRF or the segment of the arterial tree examined may partly explain the inconsistent findings. For example, while we used maximal exercise testing on a cycle ergometer with and without respiratory gas analysis, other studies showing inverse associations of CRF with indicators of arterial structure and arterial stiffness have used submaximal exercise testing and estimated VO_2peak_ or the results of indirect field tests of endurance performance [20–22]. Our approach to scale W_max_ and VO_2peak_ by LM may explain the weak association between CRF and cIMT in our study. Farr and coworkers found that higher VO_2peak_ scaled by whole body mass, but not fat-free mass, was associated with lower carotid-ankle PWV in children [26]. Moreover, Pahkala and coworkers [46] found inverse associations of estimated VO_2peak_ from a maximal cycle ergometer exercise test with aortic IMT and aortic elasticity but not with cIMT or carotid artery elasticity. Similarly, Ferreira and coworkers observed that increases in allometrically scaled VO_2peak_ from adolescence to the age of 36 years had a stronger positive association with brachial diameter and femoral artery compliance than with carotid artery distensibility at age 36 [47]. In our study, the positive association between VO_2peak_/LM and cIMT in a general population of children followed up until adolescence was largely explained by BF% and SBP. An increase in cIMT may reflect adaptive remodelling of the artery wall due to the thickening of the tunica media layer to preserve wall stress under physical strain [48] rather than atherosclerotic process in youth [7, 49–51]. The adaptive remodelling may be caused by exercise-induced shear stress on arterial walls resulting in an increase in cIMT. This phenomenon has been shown in healthy male adults [52]. However, more research is warranted to be able to confirm the existence of this phenomenon also in children and adolescents. It is worth noting that vigorous exercise training has been positively correlated with cIMT in adolescents [52], which supports the adaptive remodelling theory. Moreover, Ferreira and Gill have suggested that increases in indicators of arterial stiffness, such as PWV, reflect early arterial ageing, while increases in cIMT reflect alterations in arterial architecture and early signs of atherosclerosis [7]. They also reported that cIMT is not related to arterial stiffness in children, which is consistent with our results [7]. In conclusion, CRF in childhood and adolescence in a general population is likely to have minor effects on cIMT and carotid stiffness, adaptive arterial remodelling is a normal process in youth, and the adverse effects of poor CRF on indicators of early atherosclerosis emerge later in life.

Lower handgrip strength has been associated with higher PWV [52], pre-clinical as well as clinical atherosclerosis [53] in adults and with pre-clinical atherosclerosis in children when assessments were based on cIMT [30]. However, we found that better sit-up performance was associated with several indicators of arterial stiffness in adolescence, whereas neither handgrip strength nor standing long jump performance was related to arterial structure or stiffness in youth. Nevertheless, the associations of sit-up performance with the indicators of arterial stiffness were mainly explained by BF% and puberty status. These findings are plausible as higher adiposity has been associated with higher PWV [54] and cIMT [55] and with lower sit-up performance [56] in youth. Seemingly only Melo and coworkers have found an inverse association between muscular fitness assessed by handgrip test and cIMT [30], while Weberrus and her research group found no associations between muscular fitness and motor fitness assessed by a Fitnessgram test battery and arterial indicators such as cIMT and distensibility [20]. Advancing previous mixed results from cross-sectional studies, our longitudinal results provide new insight into existing knowledge about the association of muscular fitness and arterial structure and stiffness as we found a consistent association of better sit-up performance with lower arterial stiffness.

The strengths of this study include a well-characterised population-based sample of children followed up for eight years until adolescence and valid and reproducible indicators of various components of physical fitness and arterial structure and stiffness. We were also able to control for several possible confounding factors such as pubertal status, BF% assessed by dual-energy X-ray absorptiometry, and SBP. However, this study also has some limitations. Although we have used a validated testing protocol for CRF throughout the 8-year follow-up, the chosen protocol may not have been optimal for all participants at the 8-year follow-up due to the small increases in workload during the exercise test [47]. However, all participants at the 8-year follow-up reached the recommended levels of secondary criteria for maximal effort during exercise tests, including the mean respiratory exchange ratio of 1.1 (SD 0.06), the mean maximal heart rate of 197 beats/min (SD 8.1 beats/min), and the maximality of the exercise test evaluated by the physician supervising the test. Furthermore, arterial structure and stiffness were assessed only in adolescence which did not allow us to analyse the associations between changes in physical fitness and arterial structure and stiffness from childhood to adolescence.

Moreover, the findings of our study in a general population of mainly normal-weight children followed up until adolescence may not be generalisable to pediatric patients or youth with a higher prevalence of overweight and other cardiometabolic risk factors. Although the research protocols and conditions were kept similar at each time point, random changes in measurement conditions may have had a minor impact on the measurement results. Nor can we exclude the potential operator-related variability in skill-demanding measurements such as ultrasonography. While the attrition rate from baseline to 8-year follow-up was 45%, the participants completing the follow-up did not differ from those who dropped out other than by BF% and parental education. However, individuals with higher adiposity and lower socioeconomic backgrounds may be more prone to drop out from long-term follow-up studies, which may cause selection bias in the results. Therefore, it is important to find ways to improve adherence in intervention and follow-up studies and to better understand possible obstacles and enablers to improve adherence rates in paediatric population studies.

## Conclusions

CRF, muscular fitness, and motor fitness in childhood and adolescence have weak if any associations with arterial structure and stiffness in adolescence. Although a better sit-up performance in childhood and adolescence was consistently associated with lower arterial stiffness in adolescence, these associations were largely explained by BF%. Therefore, our findings suggest that physical fitness alone may not be the best indicator to identify youth at risk of arterial stiffening or early atherosclerosis. Based on our findings, we propose that indicators other than CRF or indicators of muscular fitness and motor fitness, such as adiposity, should be used to screen children and adolescents at increased risk of CVDs. Prevention of adiposity should be addressed in interventions aiming to improve arterial health in childhood and adolescence. Further studies should investigate whether improvements in physical fitness will lead to meaningful changes in arterial structure and stiffness from childhood to adolescence and adulthood.

## Data Availability

The data are not publicly available due to research ethical reasons and because the owner of the data is the University of Eastern Finland and not the research group. The pseudonymised data are available upon reasonable request from the corresponding author.

## Abbreviations

BF%: Body fat percentage
BMI: Body mass index
CAD: Carotid artery distensibility
CAVI: Cardio-ankle vascular index
CI: Confidence interval
cIMT: Carotid intima-media thickness
CRF: Cardiorespiratory fitness
DBP: Diastolic blood pressure
PWV: Pulse wave velocity
SBP: Systolic blood pressure
